# Editorial: The Pharmacology of Kratom and Its Alkaloids

**DOI:** 10.3389/fphar.2022.878376

**Published:** 2022-03-10

**Authors:** Oliver Grundmann, Christopher R. McCurdy, Darshan Singh, Kirsten E. Smith, Marc T. Swogger

**Affiliations:** ^1^ Department of Medicinal Chemistry, College of Pharmacy, University of Florida, Gainesville, FL, United States; ^2^ Centre for Drug Research, Universiti Sains Malaysia, Penang, Malaysia; ^3^ Translational Addiction Medicine Branch, National Institute on Drug Abuse Intramural Research Program, Baltimore, MD, United States; ^4^ Department of Psychiatry, University of Rochester Medical Center, Rochester, NY, United States

**Keywords:** kratom, Mitragyna speciosa, pharmacology, toxicity, epidemiology

Kratom (*Mitragyna speciosa* Korth.) is an ethnomedicinal tree native to Southeast Asia with a long history of traditional use. During the last two decades, all indicators and metrics we have accessed suggest that kratom consumption in the United States has increased. Although there are now many self-reported motivations for kratom use, the most prominent is the self-treatment of pain, which is mediated at least in part through opioid receptors as detailed in this special issue’s comprehensive review of mitragynine and its diastereomers “The Chemical and Pharmacological Properties of Mitragynine and Its Diastereomers: An Insight Review” (Karunakaran et al.), along with new research that indicates the involvement of adrenergic and, potentially, cannabinoid receptors, as described in “Methadone, Buprenorphine, and Clonidine Attenuate Mitragynine Withdrawal in Rats” (Hassan et al.) and “Mitragynine (Kratom)-Induced Cognitive Impairments in Mice Resemble Δ9-THC and Morphine Effects: Reversal by Cannabinoid CB1 Receptor Antagonism” (Iman et al.). Many individuals are reporting success using kratom to reduce opioid use despite kratom’s own, generally milder, withdrawal syndrome. Also in this issue, data on kratom’s potential for treating other highly-prevalent substance use disorders is being examined, as shown in “Evaluation of Kratom Opioid Derivatives as Potential Treatment Option for Alcohol Use Disorder” (Gutridge et al.).

It is noteworthy that kratom remains unregulated in the United States at the Federal level while research on its medicinal, abuse liability, and toxicity profile accrues. Such research is exemplified in “Kratom abuse potential, 2021: An updated eight-factor analysis” (Henningfield et al.), which provides a critical and rigorous evaluation of kratom pharmacology and its public health relevance. The toxicity of kratom and its over 40 known alkaloids, of which mitragynine is reported to be the most abundant, require further investigation. Another review in this issue, “Kratom Alkaloids: Interactions With Enzymes, Receptors, and Cellular Barriers” (Hanapi et al.), summarizes present knowledge regarding potential interactions of kratom and its alkaloids with enzymes and receptors that may contribute to adverse effects and affect cell barrier function. Developmental toxicity and teratogenicity appear to be distinctly different from classical opioids such as morphine, as detailed in “Comparative Toxicity Assessment of Kratom Decoction, Mitragynine and Speciociliatine Versus Morphine on Zebrafish (Danio rerio) Embryos” (Damodaran et al.). There have also been isolated cases of cardiovascular toxicity linked to kratom use, a topic further explored in “Assessment of Cardiovascular Functioning Among Regular Kratom (Mitragyna speciosa Korth.) Users: A Case Series” (Leong Bin Abdullah and Singh). Relatedly, another review, “The Adverse Cardiovascular Effects and Cardiotoxicity of Kratom (Mitragyna speciosa Korth: A Comprehensive Review” (Leong Bin Abdullah and Singh) provides insights into the currently available literature on kratom cardiotoxicity.

The use of kratom to mitigate withdrawal symptoms from an opioid use disorder (OUD) appears to correlate with its increased availability and in recognition that such use has been well characterized in Southeast Asia, as described in “Kratom Use Within the Context of the Evolving Opioid Crisis and the COVID-19 Pandemic in the United States” (Prozialeck et al.). Another prominent applicability of kratom reported by users is in the self-treatment of mood and anxiety problems. Interestingly, some of the pathways involved in antidepressant effects are also involved in addiction, most notably the expression of ΔFosB. However, while kratom did show antidepressant and analgesic activity in rats in a new research study, it did not affect ΔFosB, suggesting a differential antidepressant and analgesic mechanism (see “The Antidepressant-Like and Analgesic Effects of Kratom Alkaloids are accompanied by Changes in Low Frequency Oscillations but not ΔFosB Accumulation”) (Buckhalter et al.).

Much of the current clinical knowledge about kratom comes from either case reports or large surveys conducted among its regular users. In this issue, “Kratom Use in the United States: Both a Regional Phenomenon and a White Middle-Class Phenomenon? Evidence From NSDUH 2019 and an Online Convenience Sample” (Rogers et al.) presents survey findings from two national convenience samples with kratom-use histories that describe subgroups of users and speculates about demographic and psychosocial factors associated with use that may be changing as kratom products, media, and marketing strategies diversify. Results indicate that kratom is primarily used by White, younger, employed, and middle-class consumers, but without clear regional trends yet established. Another survey comprised of current and former kratom users, “Searching for a signal: Self-reported kratom dose-effect relationships among a sample of United States adults with regular kratom use histories” Smith et al. corroborates prior work indicating that kratom’s withdrawal severity has a link with kratom intake (dose). The survey found that kratom acute effects typically begin within minutes, but last for hours, and that effects were reported as largely compatible with and even helpful in meeting daily roles and obligations.

Finally, the increasing use of kratom in the United States and globally requires healthcare professionals to gain sufficient knowledge of this plant and products derived from it in order to facilitate productive clinical conversations. Many healthcare professionals who may encounter patients or clients using kratom do not have extensive training or expertise in medicinal chemistry or toxicology. As there are presently no published human experimental studies of kratom or its alkaloids, treatment providers have few resources with which to scientifically-inform their clinical understanding of kratom or their interactions with kratom-using people. Many available case reports lack essential context; information readily available on the internet may be inaccurate or decontextualized. This special issue is therefore particularly distinguished by the manuscript “Understanding Kratom Use: A Guide for Healthcare Providers” (Swogger et al.) which provides information about kratom to clinicians in easily understandable terms. This paper is a starting point for helping clinicians develop best practices until more human data on kratom are available.

We hope that this special issue provides readers with an updated overview of kratom, its alkaloids, and the people who use it and that it sparks interest among researchers and clinicians alike into this diverse and complex plant that we are only just beginning to understand.

